# T Cell Subsets in the Germinal Center: Lessons from the Macaque Model

**DOI:** 10.3389/fimmu.2018.00348

**Published:** 2018-02-26

**Authors:** Monica Vaccari, Genoveffa Franchini

**Affiliations:** ^1^Animal Models and Vaccine Section, Vaccine Branch, Center for Cancer Research, National Cancer Institute, Bethesda, MD, United States

**Keywords:** T follicular helper cell, T follicular regulatory cells, non-human primate, HIV infections, simian immunodeficiency virus, germinal center

## Abstract

Germinal centers (GCs) are organized lymphoid tissue microstructures where B cells proliferate and differentiate into memory B cells and plasma cells. A few distinctive subsets of highly specialized T cells gain access to the GCs by expressing the B cell zone–homing C-X-C chemokine receptor type 5 (CXCR5) while losing the T cell zone–homing chemokine receptor CCR7. Help from T cells is critical to induce B cell proliferation and somatic hyper mutation and to limit GC reactions. CD4^+^ T follicular helper (T_FH_) cells required for the formation of GCs and for the generation of long-lived, high-affinity B cells. Regulatory CD4^+^ (T_FR_) and CD8^+^ T cells co-localize with T_FH_ cells and keep their expansion in check, thus limiting GC reactions. A cytotoxic CXCR5^pos^ CD8^+^ T cell subset has been described in GCs in humans: although low in number, GC CD8^+^ T cells can expand rapidly during certain viral infections. Because these subsets find their home in secondary lymphoid tissues (lymph nodes and spleen) that are difficult to obtain in humans, GC–homing T cells have been extensively studied in mice. Nevertheless, significant limitations in using this model, such as evolutionary divergences between mice and humans and the lack of an optimal mouse model for certain human diseases, have prompted investigators to characterize GC–homing T cells in macaques instead. This review will focus on discoveries made in macaques, particularly in the non-human primate models of simian immunodeficiency virus and simian–human immunodeficiency virus infection. Indeed, experimental studies in these models have allowed researchers to gain insight into the relative role of follicular T cell subsets in HIV progression, virus persistence, and specific B cell responses induced by HIV vaccines. These discoveries have prompted the testing of novel approaches aimed to manipulate follicular T cells to increase the efficacy of HIV vaccines and to eliminate HIV reservoirs.

## Introduction

Effective antibody responses are crucial for preventing viral infections and are the basis for the majority of successful vaccination strategies (1). The quality of such antibodies is largely dependent on T cell–B cell interactions. In physiological conditions, T and B cells are subcompartmentalized within lymphoid lobules of lymph nodes (LNs). B cells reside within the outer cortex areas of the lobules enriched for the B cell-attracting CXCL13, while T cells express the (C-C motif) receptor 7 (CCR7) and recirculate through the paracortex and interfollicular cortex enriched in CCR7 ligands (CCL21 and CCL19). Following antigenic stimulation, a small number of activated T cells lose CCR7 expression and upregulate CXCR5, the receptor for CXCL13 (2–4). CXCR5^pos^ T cells travel toward the B cell-rich follicles in the outer cortex areas, where they interact with B cells (5–7). Two highly specialized CXCR5^pos^ CD4^+^ T cell subsets, T follicular helper (T_FH_) and T regulatory (T_FR_) cells, have been identified in B cell follicles. Activated T_FH_ cells migrate to the T–B borders and B cell follicles where they are required for the formation and maintenance of germinal centers (GCs) [reviewed in Ref. (8, 9)]. GCs are organized lymphoid tissue microstructures where B cells expand and differentiate during immune responses to appropriate pathogens or antigens (10, 11). In the GCs, T_FH_ cells support B cells class switching recombination and somatic hypermutation (SHM) (10, 12). GC reactions ultimately result in the selection of resting B cell memory cells and long-lived plasma cells producing antibodies with high affinity for the encountered antigen (13). The strong reaction occurring in the GCs needs to be tightly regulated to avoid the generation of autoantibodies and excessive inflammation (14–17). T follicular regulatory cells have recently been described as a subset of CXCR5^pos^ T regulatory cells that co-localize with T_FH_ cells, control their expansion, and modulate T_FH_ cell-driven B cell maturation, antibody class switching, and affinity maturation (14, 16, 17). CD8^+^ T cells are also part of the follicular T cell population (18–25). Recent studies have started to shed light on the role of these cells in regulating GC reactions and their interaction with T_FH_ and B cells in certain infections (19, 20, 23–25).

Because of their critical role in every step of B cell differentiation, T_FH_ cells have been the focus of intense interest in HIV infection. Indeed, aberrant B cell responses and B cell dysfunction are characteristics of chronic HIV infection (26). T_FH_ cells are infected with HIV (27–29), they accumulate in lymphoid tissues of some individuals during chronic infection (27, 28), and their ability to provide B cell help is impaired (30). Hence, HIV-associated changes in T_FH_ cells most likely affect the generation of effective B cell responses against the virus. Moreover, by homing to the GCs, T_FH_ cells escape immunological control and establish a persistent reservoir (21, 22, 31). The quest for an effective vaccine against HIV has also fueled intense interest in the biology of CXCR5^pos^ T cells and their role in GC reactions. Ideally, an HIV vaccine would induce high affinity broadly neutralizing HIV-1 antibodies capable of neutralizing multiple HIV-1 viral strains. These antibodies show remarkable levels of somatic mutation (32); henceforth, their generation is most likely highly dependent on effective T_FH_–B cell interactions in GCs.

Much of the current knowledge on the role of GC-resident T cells during HIV infection has been attained by studies performed in non-human primate (NHP) models. Macaques can be infected with the simian immunodeficiency virus (SIV) that closely mimics many aspects of HIV infection (33), giving the NHP model advantages by comparison to both rodents and humans. This review will focus on discoveries made in macaques, on how GC–homing T cells are affected during HIV/SIV infection, and on how HIV-associated changes in these cells may alter antibody responses. Strategies tested in NHP models aimed to target T_FH_ cells to eliminate HIV reservoir from GCs and to increase the effectiveness of HIV vaccine responses will also be discussed.

### Characterization of GCs in NHPs

Studies in mice have been fundamental in revealing the phenotype and function of GC-resident T cells and studying their key lineage-specific transcription factors (14, 16, 34). However, the similarity between humans and NHPs makes NHPs optimal for research on complex immunological interactions. Macaques have several advantages over rodents, and the first is that their genetic evolution more closely resembles those of humans (35, 36). For example, evolutionary divergence between the signaling pathways that shape T_FH_ cell differentiation in humans and mice has recently been discovered (37). Second, their immune system also resembles those of humans. Indeed, NHPs have been used to study fundamental aspects of immunology, including the development and maintenance of T cell memory (38), immunodominance (39), and the aging immune system (40). Third, macaques LN’s structure is more similar to humans than rodents LN’ structure (41). In macaque lobes, T cell zones and B cell follicles can be identified with equal function and cell distribution as in humans. Finally, lymphoid cells and a number of their different subtypes are also identifiable with equivalent markers and methodologies used in humans.

Germinal centers are typically few in LNs of naive animals, with very little T_FH_ cell number (31). Upon vaccination or infection, selected follicles are activated and develop into GCs. The interactions between cognate B and T cells have been reported to occur 1–2 days after antigen exposure (42–44). Studies in macaques have shown that GCs are formed in draining LNs a few days after intramuscular immunization at the same site of the delivery, while they are absent in contralateral LNs (45, 46). GCs in macaques can be readily identified as positive for the B cell marker CD20 and express high levels of the proliferation marker Ki67 (CD20^pos^ Ki67^hi^). Alternatively, Hoechst staining of nuclei is used to discern GCs from the adjacent marginal zone by less intense staining in immunohistochemistry analyses (47). Marginal zone B cells, responsible for an early antibody response to blood-borne pathogens, have been identified in cynomolgus and rhesus monkeys as B cells (CD19^+^, CD20^+^) expressing high levels of complement receptor 2 (CD21) and low levels of CD27 (47, 48). CD4, CD20, PD-1, and Ki67 markers were simultaneously used to study B follicles and T_FH_ cells in rhesus macaques (48).

Compared to human subjects, the use of NHPs allows researchers to conduct controlled challenge experiments, multiple live surgeries, and invasive and terminal experiments, ultimately granting access to different tissues to an extent that is not feasible in humans (49). NHP models have also been used to test sampling techniques aimed to study the cellular composition of GCs. While in macaques it is possible to surgically remove the draining LNs at different time points after an immunization or an experimental infection, this procedure is invasive and may disrupt ongoing immune responses. Two studies have used fine-needle aspirations (FNAs) technique to collect cells from LNs of pigtail and rhesus macaques (50, 51). In both models, T_FH_ cell where readably measurable, suggesting that FNA may be an interesting alternative to collect small numbers of GC cells for further analyses, while maintaining ongoing immune responses.

### Characterization of T_FH_ Cells in Macaques

T_FH_ cells in macaques have a phenotype comparable to that of humans. T_FH_ cells are considered a distinct cell subset with a specialized function and a specific transcription factor that differs from other T helper cell subsets. The master regulator of T_FH_ cell differentiation is the transcription factor Bcl-6 (52, 53). While Bcl-6 is the unique marker of T_FH_ cells, other canonical markers used to identify them are CXCR5, PD-1, and the inducible T cell costimulator, ICOS. High expression of PD-1 has been considered an effective way to identify GC–T_FH_ cells in intact tissues (31) when GCs are co-stained. In healthy macaques, PD-1^hi^ cells within the GCs are almost exclusively CD4^+^ T cells (31). Different combinations of markers have been used to define T_FH_ cell in cell suspensions by flow cytometry, in human and macaques (Table 1). The percentage of T_FH_ cells frequency in LNs depends on the choice of the markers used to define and the gating strategy. Because of the unavailability of a cross-reactive antibody for CXCR5, T_FH_ cells were originally identified in secondary lymphoid tissues of pigtail macaques as CD4^+^ T cells-expressing programmed cell death 1 of PD-1^hi^ and low levels of interleukin-7 receptor alpha (IL-7Rα) chain (CD127) (50, 54). This cell population was only present in spleen and LNs, but not in blood, and expressed high levels of ICOS and Bcl-6. In rhesus macaques, T_FH_ cells were first identified in cell suspension from LNs as central memory (CD28^hi^ CD95^hi^) CD4^+^ T cells expressing low levels of CCR7^lo^ and high levels of PD-1 and ICOS (55). When the cross-reactive anti-CXCR5 antibody clone MU5UBEE became available, co-expression of CXCR5, coupled with high levels of PD-1 expression, has been widely used to identify and sort T_FH_ cells (56–59). However, others have reported changes in both markers, and particularly in PD-1 following *ex vivo* HIV infection, warning against using only these two markers to define T_FH_ cells (60).

**Table 1:** Markers to define TFH cells in cell suspension in humans and macaques.

Species	Tissue	Infection/treatment	TFH definition	Reference
Indian rhesus macaques	LN	SIVmac251	CD28^hi^ CD95^hi^ CCR7^low^ PD-1^hi^	(55)

Pigtail macaques	LN, spleen	SIVmac239/SIVmac251	CD8^−^ CD45RA^−^ PD-1^hi^ CD127^low^	(50, 54)

Indian rhesus macaques	LN	SIVmac239	CXCR5^+^ PD-1^+^	(31)

Indian and Chinese rhesus macaques	LN, spleen	SIVmac251	CXCR5^+^ PD-1^hi^	(61)

Indian rhesus macaques	LN	SIVmac251	CD95^+^ FOXP3^−^ CXCR5^+^ PD-1^hi^	(57)

Indian rhesus macaques	LN	SIVsmE660	CXCR5^+^ PD-1^hi^ FOXP3^−^	(58)

Indian rhesus macaques	LN	SIVmac251, SHIV	CXCR5^+^ PD-1^hi^	(62)

Indian rhesus macaques	LN	–	PD-1^hi^ (enriched TFH)	(56)

Humans	LN	HIV+	CXCR5^+^ PD-1^hi^	(28)

Humans	LN	HIV+, HIV + ART, and LTNP	CD45RA^−^ CXCR5^+^ PD-1^+^ BCl-6^+^	(27)

Humans	Spleen	HIV+	CD45RA^−^ CCR7^−^ CXCR5^+^ PD-1^+^	(29)

Humans	LN	HIV+	CD45RA^−^ CXCR5^hi^	(30)

Humans	Blood	–	CD45RA^−^ CXCR5^hi^	(63)

Humans	Blood	HIV+	CXCR5^+^ CXCR3^−^ PD-1^+^	(64)

*ART, antiretroviral treatment; LN, lymph node; SHIV, simian–human immunodeficiency virus; SIV, simian immunodeficiency virus; TFH, T follicular helper cell*.

Macaques have been a useful model for validating circulating biomarkers of GC responses that can be easily translated to humans. One example is the measurement of the level of plasma CXCL13. In macaques, CXCL13 is detectable in plasma, it increases following immunization, and its levels are associated with the frequency of T_FH_ cells in LNs (65). Importantly, a substantial frequency of CD4^+^ T cells expressing CXCR5 is also present in the blood of rhesus macaques, as is the case in humans (63). Phenotypically, circulating T_FH_ (cT_FH_) cells share common markers with GC-resident T_FH_ cells and can be identified as CXCR5^pos^ PD-1^pos^ CD4^+^ T cells. However, cT_FH_ cells express lower levels of ICOS and of the activation marker CD69 than GC T_FH_ cells, suggesting that they are present in a resting phase (66). While the origin of cT_FH_ cells is still unclear, the marker expression and ability to interact with B cells and promote B cell responses *in vitro* suggest that they may be circulating counterparts of T_FH_ cells in LNs. In mice, humans, and macaques, circulating CXCR5^pos^ PD-1^hi^ CD4^+^ T cells are heterogenic and can be divided into subsets based on their expression on (C-X-C motif) chemokine receptor 3 (CXCR3), a marker for CD4^+^ T helper type 1 (Th1) cells, alone or together with CCR6. CXCR5^pos^ CXCR3^neg^ PD-1^pos^ T_FH_ cells present the most genetic and functional similarities to T_FH_ cells in LNs (64). When the expression of CCR6 is considered, cT_FH_ cells can be further divided into three subpopulations that mirror the unique phenotype and cytokine signature of lineages of non-T_FH_ CD4^+^ T cells in blood: T_FH_ type 1 (CXCR3^pos^ CCR6^neg^), type 2 (CXCR3^neg^ CCR6^neg^), and type 17 (CXCR3^neg^ CCR6^pos^). More studies are needed to identify the role of these cell subsets in generating or maintaining antibody responses to pathogens.

Functionally, T_FH_ cells help B cells by secreting cytokines and expressing surface molecules and providing survival, proliferation, and differentiation signals [reviewed in Ref. (9, 67).]. In macaques, as in humans, GC-resident T_FH_ cells express the costimulatory receptor ICOS, the costimulatory protein CD40L required for B cell survival, and they produce the B cell helper cytokines IL-21 and IL-4 although T_FH_ cells can also produce other cytokines depending on the stimulus they receive (9). IL-21 signaling is pivotal for B cell differentiation and for the development of B cell memory. *In vitro* IL-21 production is often used as a means to measure antigen-specific responses, particularly following immunization in humans (68) and macaques (69). However, T_FH_ and cT_FH_ cells produce limited quantities of IL-21. As a result, the tracking of antigen-specific responses by intracellular staining is challenging. A recent study has used the macaque model to develop a cytokine-independent technique aimed improve the quantification of antigen-specific T_FH_ cells. Havenar-Daughton et al. have shown that the co-expression of OX40 and CD25 surface markers is sufficient to identify antigen-specific GC T_FH_ and pT_FH_ cells in the LNs and blood of immunized animals (70). Importantly, this technique offers the possibility to isolate antigen-specific T_FH_ cells by cell sorting, which is not possible with intracellular cytokine detection.

### HIV-/SIV-Associated Changes in T_FH_ Cells

HIV infection is associated with numerous B cell anomalies (26). Untreated HIV and AIDS patients develop profound B cell dysfunction, characterized by hypergammaglobulinemia, and polyclonal B cell activation (26, 71–73). The majority of HIV-infected individuals and SIV-infected macaques fail to produce protective antibodies against HIV/SIV and low-affinity B cells mature inappropriately into plasma cells (74). Because T_FH_ cells are required for the induction of high-affinity antibody responses and the generation of long-lived B cell memory (75), several groups have investigated HIV/SIV-associated changes in T_FH_ cells and their possible effect on B cell abnormalities.

Recent data suggest that GC–CXCR5^+^ PD-1^hi^ T_FH_ cells are susceptible to HIV-1/SIV infection (27, 28, 54, 55, 60). Interestingly, unlike non-T_FH_ CD4^+^ T cells, T_FH_ cell frequency and number increase in chronic HIV/SIV infection in the LNs of some humans (27, 28) and macaques (31, 54, 55, 57, 58, 60). In both macaques and humans, the increase in T_FH_ cell frequency in chronic infection is approximately 10 times compared to non-infected levels (28, 55). In humans, a median of 60% of HIV-1 RNA-producing cells was found within lymphoid follicles by *in situ* hybridization in chronically infected untreated patients with a median of 17% of follicles tissues per inguinal LN (22).

Remarkably, in all the studies reported, T_FH_ cell expansion is observed only during chronic infection, but not in acute infection. Although the reason for the increase in T_FH_ cell levels during chronic HIV/SIV infection is not clear, different hypotheses have been proposed. The accumulation of T_FH_ cells during chronic SIV and HIV has been associated with immune activation (55) and plasma viremia (57, 58) in some studies. Other studies suggest that this expansion may be driven by prolonged T cell receptor stimulation (62). In mice, LCMV infection redirects CD4^+^ T cell development away from the Th1 cell responses induced during an acute infection toward T_FH_ cells (76). Others have shown that HIV-specific GC–T_FH_ cells, particularly against the gag, also expanded in chronic infection in humans (27, 28). Finally, effective antiretroviral treatment (ART) decreases the number of T_FH_ cells in humans and macaques, suggesting that active HIV replication is necessary for T_FH_ cell expansion (27, 28).

The increase in T_FH_ cells levels is also associated with increased frequency of activated GC B cells and SIV-specific antibodies (55) in macaques, and plasma cells and immunoglobulin levels in HIV infection (28). Moreover, broadly neutralizing antibodies (bNabs) are present in HIV patients with high levels of circulating CXCR5^pos^ CXCR3^neg^ PD-1^hi^ CD4^+^ T cells (64). These results suggest that T_FH_ cells may be highly functional during HIV/SIV infection; however, other studies have revealed that they provide inadequate help to B cells (30, 77). GC-resident T_FH_ cells isolated from HIV-infected patients produce less IL-21, a cytokine pivotal for GC formation, GC B cell proliferation, and B cell maturation (9). The replenishment of exogenous IL-21 *in vitro* to T_FH_/B cell co-cultures or the *in vivo* administration to SIV-infected macaques significantly improves memory B cell levels (30, 78), suggesting that lost IL-21 production may be a contributing factor to the generation of defective memory B cell responses. T_FH_ cells express a number of molecules that restrain them from excessive proliferation such as PD-1 (53). The PD-1 expression is highly increased in HIV-infected CD4^+^ T cells (31), and the level of its ligand PDL-1 on B cells increases in HIV patients. Interestingly, by blocking the PD-1–PD-L1 interaction, IL-21 production by T_FH_ cells is recovered and B cell functions are restored. Therefore, it is possible that T_FH_ cell impairments may be, at least in part, mediated by HIV-associated changes affecting B cells (30).

Studies in monkeys have reported that during SIV infection, T_FH_ cells express non-characteristic transcriptional factors together with canonical ones and that gene and cytokine expressions are skewed toward CD4^+^ Th1 cells and interferon (IFN)-γ (78). During chronic SIV infection, IFN-γ-induced genes are upregulated while the expression of the IL-4 gene is downmodulated (55). Accordingly, the majority of GC–T_FH_ cells in chronically infected macaques are positive for CXCR3^+^ and produce IFN-γ (Th1-type cytokine) alongside IL-21. While these cells are capable of helping B cells *in vitro*, they express higher levels of CCR5 and harbor more SIV-DNA than CXCR3^neg^ GC–T_FH_ cells (79). T-bet, the transcriptional regulator of Th1, is also increased in T_FH_ cells isolated from SIV-infected macaques’ spleens (61). Importantly, an association between IFN-γ^low^ IL-21^hi^ GC resident T_FH_ cells and the broad neutralization activity against the envelope was found in simian–human immunodeficiency virus (SHIV)-infected macaques (80). Taken together, these studies suggest that, while functional, T_FH_ cells may undergo changes in levels and function that may affect their ability to help B cells induce high-quality antibodies. These conclusions are corroborated by the lack of responsiveness to other infections or vaccines observed during late HIV/SIV infection.

### T_FR_ Cells and SIV-Associated Changes in Macaques

T follicular regulatory cells control the magnitude of GC reactions and avoid the onset of some autoimmune diseases (14–17). The frequency of T_FR_ cells is low in mice, humans, and monkeys compared to other CD4^+^ T cells subsets, and as for the T_FH_ cells, the percentage varies depending on the markers used to identify this subset. Phenotypically, they share the canonical markers of T_FH_ cells (CXCR5, ICOS, PD-1) and T_REGS_ [FOXP3, CD25, cytotoxic T-lymphocyte-associated protein 4 (CTLA4) positive and CD127 negative] (Table 2). Functionally, T_FR_ cells produce IL-10 and TGF-β and express the inhibitory molecule CTLA4 (16). T_FR_ cells have been characterized in the LNs of rhesus macaques as FOXP3^pos^ CD25^pos^ CXCR5^pos^ CCR7^neg^ as FOXP3^pos^ CD25^pos^ PD-1^hi^ CD127^neg^ CXCR5^pos^ CD4^+^ T cells or, alternatively, as CD25^pos^ CD127^neg^ CD3^+^ CD8^−^ T cells (57, 58, 81, 82). Depending on the markers used, their frequency ranges between 2 and 5% of CD4^+^ T cells or CD8^−^ CD3^+^ T cells. We showed that an enriched population of T_FR_ cells, obtained from the LNs of macaques by isolating sorted CD25^pos^ CD4^+^ T cells migrating toward CXCL13, was capable of suppressing autologous GC–T_FH_ cell proliferation *in vitro* (58).

**Table 2:** Markers to define TFR cells in cell suspension in humans and macaques.

Species	Tissue	Infection/treatment	TFH definition	Reference
Indian rhesus macaques	LN	SIVmac251	CD95^+^ FOXP3^+^ CD25^+^ CXCR5^+^ CCR7^−^	(57)

Indian rhesus macaques	LN	SIVsmE660	CXCR5^+^ PD-1^hi^ FOXP3^+^ CD25^+^	(58)

Indian rhesus macaques	LN	SIVmac239	CD3^+^ CD8^−^ CD25^hi^ CD127^−^ CXCR5^hi^ (GC:PD-1^hi^)	(82)

Humans	Spleen	HIV+	CD45RA^−^ CCR7^−^ CXCR5^+^ PD-1^+^ FOXP3^+^ CD25^+^	(29)

Humans	LN	HIV+	CD3^+^ CD8^−^ CD25^hi^ CD127^−^ CXCR5^hi^	(81)

T_FR_ cells are essential to the control of T_FH_ cell numbers in mice (14, 16). T_FR_ cell decrease or stagnation during chronic SIV infection may contribute to the T_FH_ cell dynamic seen in HIV infection. Two macaque studies have shown that T_FR_ cells are susceptible to infection by different SIV strains: SIV_mac251_ and SIV_smE660_ (57, 58). A recent study expanded this knowledge to humans by showing that T_FR_ cells are highly permissive to infection both *ex vivo* and *in vivo* in chronic HIV-untreated patients (81). In a longitudinal study in SIV_mac251_-infected macaques, we showed that the frequency and number of T_FR_ cells significantly decreased in LNs during the chronic phase and that the reduction was associated with an increase in T_FH_ cell levels (58). These findings were corroborated by the parallel independent study by Chowdhury et al., showing changes in the ratio of T_FH_ to T_FR_ cells in favor of T_FH_ during chronic infection with SIV_smE660_ (59). Interestingly, in a cross-sectional study, Miles et al. showed an increase in the number of GC-resident T_FR_ cells in HIV-infected humans and SIV_mac239_-infected macaques. In humans, an increase in the CD4^+^ Foxp3^+^ cell count was observed when the LN area was considered to account for LN enlargement that occurs during chronic HIV infection (81). Differences in the study design (longitudinal versus cross-sectional), T_FR_ cell definition, and analyses may have contributed to the inconsistent findings in these studies.

The increase of T_FH_ cells in chronic SIV infection has been previously associated with an increase in the titers of gp120-specific antibodies with high avidity (55). Interestingly, we observed an antithetical role of T_FR_ and T_FH_ cells in the avidity of antibodies to the SIV-gp120 protein throughout the infection. T_FR_ cell levels were associated with a reduction of binding high-avidity antibodies to SIV-gp120 in all the infected animals (58). The role of T_FR_ cells in the impairment of humoral immunity during HIV infection remains to be determined. Finally, T_FR_ cells are a relatively newly discovered population, and many of the studies performed on T_FH_ cells in humans and macaques did not include markers for T_FR_ cells exclusion (Table 1). Given their changes in frequency, susceptibility to infection and function, discriminating markers for T_FR_ should be included when studying T_FH_ cells, particularly in HIV vaccine studies.

### GC-Resident CD8^+^ T Cells in Macaques

GC-resident or CXCR5^pos^ CD8^+^ T cells are present in lymphoid tissues of humans (20, 83–85) and macaques (19, 31). In fact, three decades ago, high frequencies of CD8^+^ T cells were found in inflamed lymphoid follicles in heroin addicts and HIV-related lymphadenopathy (83, 84). However, compared to T_FH_ cells, current research on the CXCR5^+^ CD8^+^ T cells is relatively scarce. Several studies suggest that CXCR5^+^ CD8^+^ T cells represent a subset of follicular cytotoxic CD8^+^ T cells and may contribute to virus control in B cell follicles (23). Indeed, follicular cytotoxic CD8^+^ T cells express granzyme A and B and perforin at higher levels than their CXCR5^neg^ counterpart (85). Interestingly, a study identified a subset of CD8^+^ T cells with the suppressive activity on T_FH_ cells in rhesus macaques’ LNs and humans’ tonsils (19). CD8^+^ with a regulatory function produce IL-10 and express high levels of CXCR5 and the homing cell adhesion molecule CD44. Together these studies suggest that GC-resident CD8^+^ T cells may be a heterogeneous cell population.

The accumulation of infected T_FH_ cells in LNs during chronic infection is a major obstacle toward eradication. Cytotoxic CD8^+^ T cells are critical for the clearance of virus-infected CD4^+^ T cells; thus, studies focused on understanding the phenotype and function of HIV/SIV-specific CD8^+^ T cells have been performed in humans and macaques. To date, there are conflicting data on the quantity of specific CXCR5^pos^ CD8^+^ T cells and their ability to clear virus-infected T_FH_ cells. Virus-specific CD8^+^ T cells are present in GCs of humans and macaques, but they may not be enough to clear the increasing population of infected T_FH_ cells (21, 22), may be functionally impaired/exhausted (25), may exert regulatory instead of cytotoxic function, or be predisposed to provide B cell help once they enter the B cell follicles (18, 19, 25). Two *in vivo* CD8 cell depletion studies have been performed in SIV-infected macaques (24, 25). Fukazawa et al. showed that SIV is restricted to CD4^+^ T cells in the B cell follicles (with a median of 95% of productively SIV-infected cells) in macaques that are naturally controlling infection (elite controller or EC), but not in animals with normal disease progression. *In vivo* depletion of CD8^+^ cells in EC macaques resulted in a temporal redistribution of productive CD4^+^ T cells in the extrafollicular area, until CD8^+^ T cells absolute count returned to normal levels (24). Li et al. showed higher levels of both follicular and extrafollicular SIV-producing cells after CD8^+^ cell depletion in normal disease progression macaques, with the greatest increase in the extrafollicular areas (8.9 versus 3.8 cells/mm^2^ average change in the follicles) (25). Although these two studies differed in the CD8 depletion protocol (repeated low dose administrations, one single high-dose bolus), both showed profound depletion in the LNs. It should be noted that *in vivo* CD8 cell depletion may have eliminated other CD8-expressing cell populations (for example, NKs).

Some CXCR5^pos^ CD8^+^ T cells with the ability to contain LCMV have been found in GCs in mice and in blood of HIV-infected patients (86), where their levels correlated with viral load. In patients with HIV, the number of virus-specific CXCR5^pos^ CD8^+^ T cell subset is inversely correlated with viral load in LNs (86). Peripheral and GC CXCR5^pos^ CD8^+^ T cells are also present in SIV-infected macaques, where their levels increase after immunization, and it is higher in macaques controlling infection than ones who do not (87). CD8^+^ T cells can still contain viral replication in chronic infections although the mechanism of this containment is largely unknown (86). Recent work by Petrovas et al. show that CD8^+^ T cell in the GC had better killing activity than non-follicular CD8^+^ T cells, despite being less polyfunctional (20). Taken together, these results suggest that CD8^+^ T cells could be an effective component of an HIV cure strategy.

### T_FH_ Cells as Privileged Latent Reservoir

Lymphoid organs constitute the first established reservoir of HIV infection. In untreated HIV patients, viral replication is found in GCs soon after and all through the duration of infection (88–90), and the free virus can be detected even during clinical latency asymptomatic phase (91, 92). Viral replication is never completely curtailed from the LNs, and it is detected in the GCs till they involute with advancing disease (93). The macaque model of HIV-1 infection has been fundamental to study B cell follicles as immune privileged sites and for extending these observations to gut-associated lymphoid tissue (21, 94, 95). Antiretroviral therapy contains viral replication; however, it fails to eliminate the virus from lymphoid tissues. A steady-state level of very low viremia has also been described among those on ART, but the exact mechanism for persistent viremia during ART is not completely understood (96). Upon ART discontinuation, viral replication rebounds, resulting in titers similar to those observed prior to treatment. Thus, it is possible that some cellular sanctuary may exist, which allow the virus to persist.

Recent data suggest that GC–CXCR5^+^PD-1^hi^ T_FH_ cells are highly susceptible to HIV-1 infection (27, 28, 60). Some studies report that T_FH_ cells are a preferentially infected by HIV/SIV (27, 57), while others report that the permissiveness of T_FH_ cells is comparable to other subsets of memory CD4^+^ T cells (54, 55). The levels of the virus entry co-receptor CCR5 expressed by T_FH_ cells varies in different studies (54, 60, 62, 97), possibly depending on the definition used to identify T_FH_ cells. The role of levels of CCR5 expression of T_FH_ cells and susceptibility to HIV/SIV infection is also not clear. While a study reports no association between the levels of co-receptor and susceptibility (60), Xu et al. used NHP to explain the apparent discrepancy between low levels of the HIV co-receptor and the heightened permissiveness to infection (97). The group identified a subset of LN-resident T_FH_ cell precursors expressing intermediate levels of PD-1 and higher levels of CCR5 than fully differentiated PD-1^hi^ T_FH_ cells and showed that in their precursor state T_FH_ cells are highly susceptible to *in vitro* SIV infection. Other parameters may also contribute to the high susceptibility to HIV/SIV infection of T_FH_ cells described in certain studies. Recent work by Ruffin et al. showed that GC–T_FH_ cells from LNs and tonsil obtained from chronically infected patients express low levels of the HIV-1 restriction factor SAMHD1 (98, 99).

Localization of T_FH_ cells within the GCs most likely contributes to their high susceptibility to HIV/SIV infection and their expansion (22, 31). The increase in T_FH_ cell permissiveness, when compared to other memory CD4^+^ T cell subsets, does not associate with their activation status or levels of HIV co-receptor expression (60). It is possible that their unique localization in the GC may play a role in the heightened susceptibility. Indeed, it has been shown that CD4^+^ T cells located in the follicles are 40 times more likely to be infected by HIV than those located outside the follicles (22). In the GCs, the virus can be transmitted by follicular dendritic cells (FDCs) that are capable of long-term antigen retention (100). FDCs trap multiple intact viral particles on the surface and efficient transmission to GC-resident CD4^+^ T cells (101–104). Indeed, FDCs act as HIV “archives” by retaining ART-resistant virus variants that are not present elsewhere (104). While this is an interesting theory, it is also expected that other molecules may be bound together with the virus, such as antibody and complement, and it is not clear how this trapped virus would serve as a source for CCR5-expressing T_FH_ cell precursors (97). Other HIV-/SIV-associated changes in cellular composition within the GCs, such as changes in T_FH_/T_FR_ cell ratio, may contribute to the accumulation of T_FH_ cells, at least in macaques (57, 58). Moreover, the paucity of virus-specific CD8^+^ T cells in the B cell follicles, when compared to extrafollicular areas in both HIV (22) and SIV infection (21), as well as cell exhaustion or malfunction, may account for the lack of clearance of infected T_FH_ cells as previously described.

Improving the ability of potent antiviral CD8^+^ T cells to traffic into B cell follicles may result in the elimination of virus reservoirs. To determine whether the expression of CXCR5 may be sufficient for CD8^+^ T cells to enter the follicles, Ayala et al. infused six SIV-infected macaques with autologous CD8^+^ T cells genetically modified to express CXCR5 (105). The engineered T cells were found in abundance within the B follicles, with some cells localized in the proximity of infected T_FH_ cells. While CD8^+^ T cells used in this study were circulating T cells that were not selected by their specificity to HIV, this study is an important step forward in the further development of strategies aimed to eliminate virus persistence in treated patients (105). Finally, a better understanding of immune cell type localization in the GCs, particularly those with the ability to eliminate infected T_FH_ cells, will be a key for designing new eradication strategies.

### Vaccine-Induced T_FH_ Cells

The goal of a vaccine is to induce long-lasting memory responses to the pathogen. HIV presents a greater challenge than other viruses, in part because it replicates in CD4^+^ T cells and induces profound deregulation of the overall immune system. Neutralizing antibodies against the autologous virus are detectable only after years from seroconversion and only 20% of infected patients develop cross-react antibodies against different gp120 regions (106–108). Although these antibodies have shown protection in non-human macaques’ models using SHIV (109, 110), the most desirable response for an HIV vaccine would be the induction of bNabs. bNabs can act against a wide spectrum of viruses by targeting relatively conserved regions on the surface HIV envelope trimer spike (111). Because of the striking amount of SHM in HIV bNabs (112–114), it is conceivable that T follicular helper cells and GCs play a critical role in generating such antibodies. However, the elicitation of bNabs trough vaccination is challenging. These antibodies are uncommon (produced by 10% of HIV-infected individuals) (115), and conventional HIV vaccines are unable to induce the number of mutations observed in bNabs. Some studies have been performed in macaques to test the ability of different adjuvants to stimulate T_FH_ differentiation, SHMs, and affinity maturation to neutralizing HIV epitopes. Importantly, in this model, the levels of GC-resident T_FH_ cells are associated with the generation of neutralizing antibody breadth during SIV/SHIV infection (80). A study in macaques revealed scarce differences in the mean SHM levels or CDR H3 lengths using eight different adjuvants in combination with a gp140 protein vaccine to immunize macaques (116). PLGA, a toll-like receptor ligand containing nanoparticles adjuvant, induces strong GCs reactions in monkeys (117, 118). A native-like Env trimer, given twice intramuscularly together with a strong adjuvant ISCOMATRIX induced neutralizing antibodies against Tier 2 (difficult to neutralize) viruses (118). Potent T_FH_ cell responses were found in LNs of rhesus macaques after immunization, and no changes were observed in the levels of T_FR_ cells. The effectiveness of this immunization was not tested in challenge experiments.

While the search for a strategy capable of bestowing protection *via* the induction of neutralizing antibodies or even bNAbs continues, non-neutralizing antibodies may also be increased during vaccination by increasing T_FH_ cell differentiation. Of note, the only vaccine to provide low, but significant protection from HIV acquisition in humans induced binding non-neutralizing antibodies to the variable region of the gp120 V2 loop (119, 120). In a retrospective study, it was shown that volunteers vaccinated with an ALVAC-SIV + gp120 alum vaccine had higher levels of IL-21-producing cT_FH_ cells than individuals immunized with strategies that failed to protect (68). Therefore, it is possible that an increase in binding antibodies to gp120, and particularly to the V2 loop, *via* T_FH_ cells may increase the efficacy of an HIV vaccine, despite the absence of neutralization. Studies in macaques have revealed that conventional vectored vaccines indeed stimulate T_FH_ cells in combination with gp120 or gp140 protein boosts. Codelivery of MVA-SIV and gp120 protein in alum increased the levels of CXCR3^pos^ CXCR5^pos^ CD4^+^ T cells in the blood and LNs of rhesus macaques measured at the peak of immune responses after vaccination. Interestingly, while CXCR3^POS^ cT_FH_ cells favored antibody responses, they were also associated with increased peak viremia upon infection with SIV_mac251_ (121). An Adenovirus 5- based vector vaccine, encoding for Env, Gag, and Nef, followed by a gp120 or gp140 protein boost induced IL-21-producing T_FH_ cells in rhesus macaques’ LNs. T_FH_ cell levels measured after 2 weeks from the last immunization were associated with the titers of binding antibodies to the gp120 (69). While this vaccine only protected female macaques from SIV_mac251_ infection, it is noteworthy that only small differences in the levels of IL-21-producing T_FH_ cells were found when animals were stratified by sex. Taken together, these studies suggest that T_FH_ cells are induced by different vaccination strategies, and their induction results in potentially protective antibody responses that are measurable in LNs and blood. Studies comparing different strategies side by side should be performed to shed light on the association between the levels and function of T_FH_ cell induction and vaccine efficacy.

## Conclusion

The NHP model has played a fundamental role in understanding the dynamics of T_FH_ cells during HIV infection and their role as major sites for viral replication and the establishment of viral reservoirs. This model has been a key to the development of new techniques to study T_FH_ cells and GC responses that can be translated to humans, and it makes it possible to conduct preclinical studies aimed at eradicating HIV. Undoubtedly a fuller appreciation for the range of cells participating in meaningful cellular reservoirs could result in a rational attack on latent HIV-1 and may provide inroads into creating an effective vaccine designed to generate HIV neutralizing antibodies. However, the obvious limitation is that NHPs are not humans. Much of what is learned from non-human primates, especially at the preclinical level, must be validate in humans.

## Author Contributions

MV drafted the manuscript. MV and GF critically revised the manuscript.

## Conflict of Interest Statement

The authors have no relevant affiliations or financial involvement with any organization or entity with a financial interest in or conflict with the subject matter or materials discussed in the manuscript.
